# Racial/ethnic differences in maternal feeding practices and beliefs at 6 months postpartum

**DOI:** 10.1017/S1368980021005073

**Published:** 2022-12

**Authors:** Tayla von Ash, Anna Alikhani, Cynthia Lebron, Patricia Markham Risica

**Affiliations:** 1 Department of Behavioral and Social Sciences, Brown School of Public Health, Box G-S121-8, Providence, RI 02912, USA; 2 Center for Health Promotion & Health Equity, Brown School of Public Health, Providence, RI, USA; 3 School of Nursing and Health Studies, University of Miami, Miami, FL, USA

**Keywords:** Racial/ethnic differences, Maternal feeding, Feeding practices, Feeding beliefs

## Abstract

**Objective:**

To examine racial/ethnic differences in maternal feeding practices and beliefs in a sample of low-income smoke-exposed women.

**Design:**

Cross-sectional analysis using data collected during a randomised control trial. Maternal feeding practices and beliefs were assessed using the Infant Feeding Questionnaire (IFQ), which was administered at 6 months postpartum. ANOVA was used to examine differences in IFQ items by race/ethnicity, while multivariable linear regression models were used to examine differences in IFQ factor scores by race/ethnicity adjusting for potential confounders.

**Setting:**

Participants were recruited from prenatal clinics.

**Participants:**

343 women (39 % non-Hispanic White, 28 % Hispanic/Latina, 13 % Black, and 20 % other).

**Results:**

Racial/ethnic minority mothers were more likely than non-Hispanic White mothers to put cereal in their infant’s bottle so that the infant would stay full longer (*P* = 0·032), state their infant wanted more than just formula or breast milk prior to 4 months (*P* = 0·019), allow their infant to eat whenever he/she wanted (*P* = 0·023) and only allow their infant to eat at set times (*P* < 0·001). Adjusting for potential confounders, racial/ethnic minority mothers had higher scores for factors 1 (concern about infant undereating or becoming underweight), 2 (concern about infant’s hunger), 4 (concern about infant overeating or becoming overweight) and 5 (feeding infant on a schedule), and lower scores for factor 7 (social interaction with the infant during feeding) than White mothers. Racial/ethnic differences were not found for the other two factors.

**Conclusions:**

Differences in maternal feeding practices and beliefs across race/ethnicity are present at 6 months postpartum.

By the time children reach preschool age, racial/ethnic disparities in obesity are present. Specifically, the prevalence of obesity is 16·5 % among Hispanic/Latinx, 11·6 % among Black, 7·0 % among Asian and 9·9 % among White American children aged 2–5 years^(1)^. Higher prevalence among racial/ethnic minorities suggests a need to examine risk and protective factors for obesity early in life. While numerous studies have identified early-life biological and behavioural factors that influence obesity risk throughout the lifespan, the literature on racial/ethnic differences in maternal feeding factors during infancy has largely focused on breast-feeding status and age of introduction to solids^(2,3)^. Compounded by the fact that the majority of early childhood obesity prevention studies in general have been conducted with higher socio-economic, non-ethnically diverse samples, there remains a knowledge gap in the field when it comes to understanding the emergence of racial/ethnic disparities in childhood obesity rates^(2,4)^.

Parental feeding behaviours, beyond those directly related to infant dietary patterns (e.g. mode of infant feeding and early introduction of solids), influence obesity risk during early life^(5,6)^. For example, when faced with concerns about child underweight or overweight, caregivers often assume more control over child nutrition^(7)^. However, the decisions parents make about feeding their children have immediate and long-lasting implications for child growth and development^(8)^. For example, maternal control of feeding can affect weight gain acceleration or deceleration in children as early as infancy^(9)^. Infants naturally regulate their energy intakes, but parents’ behaviours can override hunger and satiety cues^(10)^. Pressured feeding (i.e. encouraging infants to finish their bottle), ‘bottle propping’ (i.e. giving infants a bottle by leaning it on a pillow, blanket or other support) and giving infants a bottle to go to bed with have been independently found to be associated with increased obesity risk^(11,12)^. However, responsive feeding, generally defined as developmentally appropriate responses to the infant’s hunger and satiety cues, can have a positive effect on infant weight gain. Although feeding in the first year of life necessitates a great deal of parental control, infants do best when they are allowed to self-regulate their food consumption^(9)^.

Independent of potential confounders, Black and Hispanic/Latinx children have demonstrated increased odds of rapid infant weight gain, greater maternal control of infant feeding, bottle propping and pressured feeding compared with White children^(13,14)^. Differences in risk and protective factors have been attributed to socio-economic status, culture, acculturation and a history of disadvantage and discrimination^(3,15)^. Studies have also demonstrated how feeding styles in Black and Hispanic/Latinx parents influence infant energy intake and infant size, and how maternal characteristics can be associated with infant feeding styles^(16,17)^. Racial/ethnic differences in early-life risk factors for obesity require further understanding and evaluation as they may contribute to the high prevalence of obesity among minority preschool-age children and may thus signify important areas for intervention.

The Infant Feeding Questionnaire (IFQ), developed by Baughcum *et al.*, assesses maternal feeding practices that may promote self-regulation and other healthy eating habits in children to prevent obesity^(18)^. Specifically, the IFQ was designed to assess nurture-based feeding practices adopted by mothers when feeding their infants during the first year of life as well as the beliefs that guide such practices.

Breast-feeding is less common among low-resourced women^(19)^, who are also more likely to smoke or be smoke exposed^(20)^. The Baby’s Breath study created and tested an intervention for decreasing smoke exposure of low-income pregnant women and their newborns. The purpose of this study was to use the IFQ to examine racial/ethnic differences in maternal infant feeding practices and beliefs at 6 months postpartum in a sample at high risk for early obesity.

## Methods

### Study design

This secondary data analysis cross-sectionally examines data collected for Baby’s Breath, a randomised control trial aimed at limiting environmental tobacco smoke exposure among pregnant women in the state of Rhode Island^(21,22)^. Participants were recruited from prenatal clinics that largely serve low-income women in the Providence area. Women were eligible if they spoke English, were at least 18 years of age, had access to a working telephone and VCR/DVD player and were either a current smoker, recently quit smoker or a non-smoker with current exposure to second-hand smoke from someone in their household. Women who were pregnant with multiples or further than 16 weeks’ gestation at the time of recruitment were not eligible to participate. Additional recruitment details along with intervention details have been published elsewhere^(21)^. Briefly, participants were randomised to receive newsletters and videos during pregnancy and the first 6 months of the postpartum period aimed at either smoking cessation and avoidance or other healthy pregnancy topics. Any information regarding infant feeding was given to both experimental groups. Informed written consent was obtained from all participants, and they were compensated for their time. The analytic sample for this analysis includes participants for which maternal feeding practices and beliefs were assessed at 6-month postpartum (*n* 343).

### Measures

#### Main exposure

The primary exposure in this analysis was mother’s racial/ethnic background. At baseline (16 weeks’ gestation), participants reported whether or not they identified as *Hispanic or Latina*. They were then asked if they identified as *American Indian or Alaskan Native, Asian, Black or African American, Caucasian or White, Native Hawaiian or Pacific Islander*, multiracial and/or *other*. The Department of Finance method of racial/ethnic classification, in which Hispanic/Latinx is deemed a mutually exclusive racial category, was used, such that Hispanic/Latina mothers were classified as Hispanic/Latina regardless of their racial identity^(23)^. Given the sample distribution, we examined race/ethnicity as four-level categorical variable in our analyses, with categories non-Hispanic White, Hispanic/Latina, non-Hispanic Black and other (which included multiracial individuals).

#### Outcomes

The outcomes of interest were maternal infant feeding practices and beliefs self-reported by mothers at 6 months postpartum using the IFQ^(18)^ (see Table 1). The IFQ consists of twenty items, for which factor analysis resulted in the following seven factors: (1) concern about the child undereating or becoming underweight, (2) concern about infant’s hunger, (3) awareness of infant’s hunger and satiety cues, (4) concern about the infant becoming overweight or overeating, (5) feeding the infant on a schedule, (6) using food to calm the infant’s fussiness and (7) social interaction during feeding. Items are assessed using a 5-point Likert scale. The response options for the 12 items assessing maternal feeding practices or child eating behaviours include 0 = never, 1 = rarely, 2 = sometimes, 3 = often and 4 = almost always. The response options for the remaining eight items, which assess maternal beliefs, are 0 = disagree a lot, 1 = disagree a little, 2 = no strong feelings either way, 3 = agree a little and 4 = agree a lot. Higher scores thus indicate a higher frequency of the specified maternal feeding practice or a higher agreement with the maternal belief statement. In addition to the continuous scales, we also dichotomised the items to report the proportion of mothers who commonly (i.e. sometimes, often or almost always) engaged in each practice and agreed (either a little or a lot) with each belief statement.

**Table 1:** Items in the Infant Feeding Questionnaire

Maternal feeding practices/child eating behaviours*	Factor†
1. Since your baby was born, how often have you let your baby eat whenever he or she wanted to?‡	5
2. How often have you worried that your baby was eating enough?	1
3. How often have you only allowed your baby to eat at set times?	5
4. When your baby became fussy, how often was feeding him/her the first thing you have done?	6
5. How often have you worried that he/she was eating too much?	4
6. How often has it been a struggle to get your baby to eat?	1
7. How often have you become upset if your baby was eating too much?	4
8. How often have you talked or sang to your baby while you fed him/her?	7
9. How often have you put infant cereal in your baby’s bottle so he/she would sleep at night?	2
10. How often have you held your baby when giving him/her a bottle?	7
11. When your baby was less than 4 months old, how often did he/she want more than just formula and/or breast milk?	2
12. How often have you put cereal in your baby’s bottle so he/she would stay full longer?	2
Maternal beliefs§	
13. How often have you felt that if you didn’t encourage your baby to eat, then he/she wouldn’t eat enough?	1
14. Feeding your baby is the best way to stop his/her fussiness	6
15. You know when your baby is hungry	3
16. You are worried that he/she will become underweight	1
17. You know when he/she is full	3
18. He/she knows when he/she is hungry	3
19. You worry that he/she will become overweight	4
20. He/she knows when he/she is full	3

* Response options were 0 = never, 1 = rarely, 2 = sometimes, 3 = often, 4 = almost always.

† Factors are: (1) concern about the child undereating or becoming underweight, (2) concern about infant’s hunger, (3) awareness of infant’s hunger and satiety cues, (4) concern about the infant becoming overweight or overeating, (5) feeding the infant on a schedule, (6) using food to calm the infant’s fussiness and (7) social interaction during feeding.

‡ Item reverse scored when calculating factor score.

§ Response options were 0 = disagree a lot, 1 = disagree a little, 2 = no strong feelings either way, 3 = agree a little, 4 = agree a lot.

Consistent with Baughcum *et al.*, factor scores were created by calculating the mean score of the items included in each factor^(18)^. If any item was missing for factors consisting of just two or three items (i.e. factors 2, 4, 5, 6 and 7), the factor score was considered missing. Factors with four items (i.e. factors 1 and 3) were considered missing if more than one item was missing; if only one item was missing, the missing item score was replaced with the mean score of the other items in that factor before calculating the factor score. Also consistent with Baughcum *et al*., the first item ‘Did you let him/her eat whenever he/she wanted to?’ was reverse scored because of negative loading on factor 5 (feeding infant on a schedule)^(18)^. Cronbach’s *α* scores for these factors in the Baby’s Breath sample were similar to those reported by Baughcum *et al.* (2001)^(18)^.

#### Other measures

Socio-demographic and other sample characteristics were collected at baseline via survey, with the exception of parity, which was abstracted from hospital records. Participant age was reported as a continuous variable while all other variables were categorical. Marital status was dichotomised as either married/cohabitating or not married/cohabitating. Parity was dichotomised as either multiparous (previous live births) or not. For employment status, participants indicated whether they were employed full time, employed part time, not employed, a student or other. Participants reported their highest level of education which was categorised as less than high school diploma, high school diploma or general education degree, or at least some college or technical school. As this was a low-income sample, annual household income was categorised as less than $10 000 per year, between $10 000 and $30 000 per year or more than $30 000 per year. Finally, we assessed breast-feeding status in the postpartum period via survey by asking participants if they had initiated breast-feeding after birth, as well as whether or not they were exclusively breast-feeding, exclusively formula feeding or mixed feeding (i.e. partially breast-feeding) at both 12 weeks and 6 months postpartum.

### Statistical analyses

Bivariate associations between race/ethnicity and socio-demographic characteristics and breast-feeding status were analysed using *χ* ^2^ tests (with Fisher’s exact tests used when there were cell values less than five), while associations with continuous scores from the IFQ were analysed using ANOVA. We then constructed multivariable regression models to assess associations between racial/ethnic background with IFQ factor scores adjusting for potential confounders. Potential confounders, informed by the literature, included age, marital status, parity, education, household income and breast-feeding status. Multiple imputation was used for missing covariate data which was assumed to be missing at random. The statistical significance level was set at 0 05 for all procedures, with analyses performed using Stata 15.0 (*Stata: Software for Statistics and Data Science*, 2020).

## Results

The racial/ethnic composition of the sample was 39 % non-Hispanic White (*n* 134), 28 % Hispanic/Latina (*n* 95), 13 % Black (*n* 46) and 20 % other (*n* 68). More than half (56 %) of the mothers who were classified as other identified as multiracial; American Indians/Alaskan Natives (22 %) and Asians (16 %) were also represented in this group (See Table 2). The average age of participants at baseline was 24·0 (sd 5·0) years, just over half (53 %) of the participants were married or cohabitating and half (51 %) had no prior children. Employment status and highest level of education varied across the sample, with 47 % of participants not employed and 37 % not having received a high school diploma. Three-quarters of the sample (74 %) had an annual household income of $30 000 or less, with 42 % of the overall sample having an annual household income less than $10 000. This distribution slightly over-represents non-White populations in comparison to the overall population in 2015 as 57 % non-Hispanic White, 7 % non-Hispanic Black, 25 % Hispanic, 12 % other or mixed race exclusive of these groups. (PRAMS) and 13 % with less than 12 years of education, though the proportion married was similar^(24)^.

**Table 2:** Sample demographics, overall and by race/ethnicity

	Overall		Non-Hispanic White		Hispanic/Latina		Black		Other*		*P*-value
	*n*	%	*n*	%	*n*	%	*n*	%	*n*	%	
Sample size	343	100	134	39	95	28	46	13	68	20	
Age											0·278
<21	104	30	44	32	35	37	9	20	16	24	
21–25	128	37	49	36	32	34	17	37	30	44	
26–35	101	29	36	27	25	26	20	34	20	29	
>36	10	3	5	4	3	3	0	0	2	3	
Marital status											0·038
Married/cohabitating	180	53	79	60	46	48	17	37	38	57	
Not married/cohabitating	160	47	53	40	49	52	29	63	29	43	
Multiparous (previous live births)											0·017
No	163	51	79	62	37	44	20	44	27	44	
Yes	155	49	48	38	47	56	25	56	35	56	
Employment status											0·207
Full time	75	22	23	18	22	23	15	33	15	22	
Part time	77	23	30	23	21	22	14	30	12	18	
Not employed	161	47	66	50	49	52	14	30	32	47	
Student	12	4	6	5	2	2	1	2	3	4	
Other	17	5	8	6	1	1	2	4	6	9	
Highest level of education											0'.437
< High school diploma	127	37	45	34	32	34	17	37	33	49	
High school diploma/GED	120	35	50	38	37	39	16	35	17	25	
Some college/tech. school	93	27	37	28	26	27	13	28	17	25	
Annual household income											0.200
<$10 000	145	42	52	39	47	49	19	41	27	39	
$10 000–30 000	109	32	38	28	30	32	15	33	26	38	
>$30 000	42	12	22	16	5	5	6	13	9	13	
Do not know/refused	47	14	22	16	12	14	6	13	6	9	
Breast-feeding status											
Initiated breast-feeding	209	61	71	53	75	79	30	65	33	49	<0.001
Breast-feeding at 12 weeks											0.066
Exclusive	19	6	13	10	2	2	1	2	3	4	
Partial	56	16	16	12	22	23	9	20	9	13	
Breast-feeding at 6 months											0.018
Exclusive	10	3	9	7	0	0	1	2	0	0	
Partial	23	7	6	4	10	11	4	9	3	4	

Ns may not add to the total sample size due to missing values; GED, general education degree.

* Includes fifteen individuals identifying as American Indian/Alaskan Native, eleven Asian, one Native Hawaiian or Other Pacific Islander, three other and thirty-eight multiracial; *P*-value is for *χ* ^2^ test, Fisher’s exact used when there were cell values <5.

Demographic differences were observed across race/ethnicity with Hispanic/Latina and Black participants less likely to be married or cohabitating and more likely to be multiparous compared with non-Hispanic White children. Differences across race/ethnicity were also observed for breast-feeding status. While more than half of the sample (61 %) initiated breast-feeding after birth, initiation was highest among Hispanics/Latinas (79 %, *P* < 0·001) compared with the other groups. However, by 12 weeks only 22 % of the sample was still breast-feeding, with only 6 % of the sample exclusively breast-feeding and no differences by race/ethnicity. By 6 months, only 10 % of the sample was still breast-feeding (3 % exclusively). Of the mothers exclusively breast-feeding at 6 months, all but one was non-Hispanic White.

Table 3 displays the sample averages for maternal feeding practices and behaviours at 6 months as well as differences in means across racial/ethnic categories. While nearly half of the mothers in this sample (48 %) reported (either sometimes, often or almost always) worrying about whether or not their infant was eating enough, only 11 % of mothers worried that their infant would become underweight. Mothers commonly endorsed at least sometimes, often or almost always putting cereal in their infant’s bottle so that they would stay full (46 %) or sleep longer (43 %), and half (48 %) of the sample reported at least sometimes, often or almost always feeling that their infant wanted more than just formula and/or breast milk prior to being 4 months old. The large majority of mothers (92–99 %) felt agreed that both they and their infant knew when he or she was hungry and full. However, 37 % at least sometimes, often or almost always worried that their infant was eating too much and 22 % agreed with the statement that they worried that their infant would become overweight. Most mothers at least sometimes, often or almost always allowed their infant to eat whenever he or she wanted (84 %) and reported that at least sometimes, often or almost always feeding was the first thing they did when their infant was fussy (76 %). Most mothers also reported that they at least sometimes, often or almost always held their infant when giving a bottle (96 %) and at least sometimes, often or almost always talked to him or her while feeding (93 %).

**Table 3:** Maternal feeding practices and beliefs at 6 months, overall and by race/ethnicity

	Overall				Non-Hispanic White		Hispanic/Latina		Black		Other*		*P*-value
	*n*	%†	Mean	sd	Mean	sd	Mean	sd	Mean	sd	Mean	sd	
Factor 1: concern about infant undereating or becoming underweight													
Worried infant was not eating enough	164	48	1·77	1·69	1·49	1·47	1·79	1·60	1·76	1·54	1·77	1·69	0·439
Struggled to get infant to eat	38	11	0·40	0·86	0·38	0·83	0·38	0·91	0·40	0·69	0·46	0·94	0·938
Worried infant would become underweight‡	31	9	0·53	0·99	0·38	0·76	0·57	1·04	0·74	1·10	0·61	1·22	0·130
Infant needed encouragement to eat enough‡	20	6	0·47	1·08	0·28	0·81	0·59	1·13	0·63	1·37	0·56	1·20	0·072
Factor 2: concern about infant’s hunger													
Cereal in bottle so infant would stay fuller longer	157	46	1·31	1·47	1·03	1·38	1·47	1·55	1·64	1·40	1·42	1·50	0·032
Cereal in bottle so infant would sleep longer at night	147	43	1·59	1·46	1·38	1·37	1·73	1·53	2·00	1·48	1·54	1·50	0·064
Before age 4 months, infant wanted more than just formula and/or breast milk	164	48	1·42	1·47	1·16	1·41	1·57	1·49	1·89	1·43	1·40	1·49	0·019
Factor 3: awareness of infant’s hunger and satiety cues													
Infant knew when hungry‡	332	97	3·93	0·32	3·93	0·34	3·97	0·18	3·93	0·33	3·88	0·41	0·399
Infant knew when full‡	314	92	3·78	0·66	3·79	0·58	3·76	0·74	3·65	0·90	3·88	0·50	0·326
I knew when infant was full‡	323	94	3·88	0·47	3·90	0·37	3·86	0·59	3·84	0·52	3·88	0·41	0·916
I knew when infant was hungry‡	338	99	3·65	0·87	3·64	0·90	3·74	0·73	3·53	1·04	3·62	0·90	0·589
Factor 4: concern about infant overeating or becoming overweight													
Worried infant was eating too much	126	37	1·16	1·31	1·01	1·24	1·25	1·38	1·37	1·45	1·19	1·26	0·347
Worried infant would become overweight‡	74	22	0·30	0·72	0·22	0·62	0·30	0·73	0·33	0·73	0·46	0·87	0·170
Upset if infant ate too much	28	8	0·95	1·36	0·81	1·23	1·07	1·50	1·15	1·49	0·91	1·31	0·361
Factor 5: feeding infant on a schedule													
Allowed infant to eat whenever wanted§	284	84	2·93	1·31	2·20	1·22	2·71	1·36	2·72	1·29	2·85	1·37	0·023
Only allowed infant to eat at set times	183	54	1·83	1·58	1·26	1·45	1·76	1·54	2·09	1·70	2·20	1·49	<0·001
Factor 6: using food to calm infant’s fussiness													
Feeding infant was the best way to stop fussiness‡	84	25	2·19	1·20	2·19	1·14	2·26	1·29	2·24	1·27	2·09	1·18	0·847
When fussy, feeding infant was first thing you would do	259	76	1·60	1·34	1·57	1·22	1·72	1·44	1·57	1·28	1·53	1·45	0·804
Factor 7: social interaction with the infant during feeding													
Talked or sang to infant while feeding	320	93	3·25	1·02	3·37	0·89	3·16	1·09	3·26	0·88	3·16	1·23	0·393
Held infant while giving a bottle	324	96	3·53	0·91	3·64	0·79	3·43	1·04	3·53	0·87	3·43	0·94	0·267

* Includes those identifying as multiracial.

† *n* (%) represents those agreeing either a little or a lot to belief statements and those who either sometimes, often or almost always practised behaviours in practice statements.

‡ Responses were recorded using the following scale: 0 = disagree a lot, 1 = disagree a little, 2 = no strong feelings either way, 3 = agree a little, 4 = agree a lot; response options for all other items were: 0 = never, 1 = rarely, 2 = sometimes, 3 = often, 4 = almost always.

§ Item was reverse scored; *P*-value is for ANOVA test.

Racial/ethnic differences in several feeding practices and behaviours were observed. Racial/ethnic minority mothers more frequently put cereal in their infant’s bottle so the infant would stay full longer (*P* = 0·032) and stated their infant wanted more than just formula or breast milk prior to 4 months (*P* = 0·019) than non-Hispanic White mothers. Allowing the infant to eat whenever he/she wanted (*P* = 0·023) was also more frequently reported by racial/ethnic minority mothers than non-Hispanic White mothers, however, so too was only allowing the infant to eat at set times (*P* < 0·001).

Table 4 shows the multivariable linear regression models for each IFQ factor. In bivariate analyses, we found racial/ethnic differences for factors 2 (concern about infant’s hunger), 5 (feeding infant on a schedule) and 7 (social interaction with the infant during feeding). These associations persisted after adjustment for maternal age, marital status, parity, education, household income and breast-feeding status at 6 months. After adjustment, Hispanic/Latina and Black mothers were more concerned about their infant’s hunger (*β*: 0·40 (95 % CI 0·09, 0·71) and *β*: 0·61 (95 % CI 0·22, 0·99), respectively) than non-Hispanic White mothers. They were also more likely to feed on a schedule (*β*: 0·70 (95 % CI 0·37, 1·02) and *β*: 0·60 (95 % CI 0·20, 1·00), respectively), as were mothers classified as other compared with non-Hispanic White mothers (*β*: 0·60 (95 % CI 0·25, 0·95)). Compared with non-Hispanic White mothers, Hispanic/Latina mothers and mothers classified as other reported less social interaction with their infant during feeding (*β*: –0·23 (95 % CI –0·43, –0·04) and *β*: –0·26 (95 % CI –0·47, –0·04), respectively).

**Table 4:** Multivariable adjusted associations of race/ethnicity and Infant Feeding Questionnaire factors

	Unadjusted *β*	95 % CI	Adjusted *β*	95 % CI
1. Concern about infant undereating or becoming underweight				
Race/ethnicity				
Hispanic/Latina	0·20	0·00, 0·40	0·25	0·04, 0·46*
Black	0·24	–0·02, 0·50	0·22	–0·04, 0·49
Other	0·22	–0·01, 0·44	0·24	0·01, 0·46*
Age			0·03	0·01, 0·05*
Married/cohabitating			−0·01	–0·17, 0·16
Multiparous			−0·13	–0·32, 0·06
Education level some college			0·01	–0·18, 0·21
Annual household income			0·00	–0·12, 0·13
Breast-feeding at 6 months				
Exclusive			0·22	–0·27, 0·72
Partial			0·09	–0·24, 0·41
2. Concern about infant’s hunger				
Race/ethnicity				
Hispanic/Latina	0·41	0·11, 070*	0·40	0·09, 0·71*
Black	0·63	0·25, 1·01*	0·61	0·22, 0·99*
Other	0·26	–0·07, 0·59	0·24	–0·10, 0·58
Age			0·02	–0·01, 0·05
Married/cohabitating			−0·03	–0·28, 0·21
Multiparous			−0·34	–0·63, –0·05*
Education level some college			0·09	–0·19, 0·38
Annual household income			−0·07	–0·26, 0·12
Breast-feeding at 6 months				
Exclusive			−1·01	–1·74, –0·28*
Partial			−0·21	–0·69, 0·27
3. Awareness of infant’s hunger and satiety cues				
Race/ethnicity				
Hispanic/Latina	0·02	–0·09, 0·13	0·02	–0·09, 0·13
Black	−0·07	–0·21, 0·06	–0·08	–0·22, 0·06
Other	0·00	–0·12, 0·12	0·00	–0·12, 0·12
Age			0·00	–0·01, 0·01
Married/cohabitating			−0·06	–0·15, 0·03
Multiparous			−0·03	–0·13, 0·08
Education level some college			0·01	–0·09, 0·11
Annual household income			0·02	–0·04, 0·09
Breast-feeding at 6 months				
Exclusive			−0·06	–0·32, 0·21
Partial			−0·05	–0·23, 0·13
4. Concern about infant overeating or becoming overweight				
Race/ethnicity				
Hispanic/Latina	0·20	–0·03, 0·42	0·25	0·01, 0·49*
Black	0·27	–0·02, 0·55	0·31	0·01, 0·61*
Other	0·17	–0·08, 0·42	0·20	–0·06, 0·46
Age			0·01	–0·02, 0·03
Married/cohabitating			0·07	–0·12, 0·26
Multiparous			−0·15	–0·37, 0·07
Education level some college			0·08	–0·14, 0·30
Annual household income			0·01	–0·14, 0·17
Breast-feeding at 6 months				
Exclusive			0·09	–0·47, 0·65
Partial			−0·17	–0·54, 0·20
5. Feeding infant on a schedule				
Race/ethnicity				
Hispanic/Latina	0·75	0·44, 1·05*	0·70	0·37, 1·02*
Black	0·65	0·26, 1·04*	0·60	0·20, 1·00*
Other	0·64	0·30, 0·98*	0·60	0·25, 0·95*
Age			0·00	–0·02, 0·03
Married/cohabitating			−0·16	–0·42, 0·10
Multiparous			−0·06	–0·36, 0·23
Education level some college			0·14	–0·15, 0·44
Annual household income			−0·07	–0·26, 0·13
Breast-feeding at 6 months				
Exclusive			−0·65	–1·41, 0·10
Partial			−0·30	–0·80, 0·20
6. Using food to calm infant’s fussiness				
Race/ethnicity				
Hispanic/Latina	0·10	–0·18, 0·38	0·21	–0·08, 0·51
Black	0·02	–0·34, 0·37	0·04	–0·32, 0·41
Other	−0·07	–0·38, 0·24	−0·03	–0·34, 0·29
Age			0·03	0·00, 0·06*
Married/cohabitating			0·08	–0·15, 0·32
Multiparous			−0·14	–0·41, 0·13
Education level some college			−0·09	–0·36, 0·17
Annual household income			0·10	–0·09, 0·28
Breast-feeding at 6 months				
Exclusive			0·45	–0·24, 1·14
Partial			−0·14	–0·60, 0·31
7. Social interaction with the infant during feeding				
Race/ethnicity				
Hispanic/Latina	−0·21	–0·40, –0·01*	−0·23	–0·43, –0·04*
Black	−0·10	–0·34, 0·14	−0·16	–0·41, 0·08
Other	−0·21	–0·42, 0·01	−0·26	–0·47, –0·04*
Age			0·01	–0·01, 0·02
Married/cohabitating			−0·10	–0·26, 0·05
Multiparous			−0·05	–0·23, 0·13
Education level some college			0·05	–0·13, 0·22
Annual household income			0·04	–0·08, 0·15
Breast-feeding at 6 months				
Exclusive			−1·15	–1·73, –0·56*
Partial			−0·35	–0·65, –0·04*

* *P* < 0 05.

Referent group for race/ethnicity is non-Hispanic White, for marital status is not married/cohabitating, for multiparous is no, for education is some college and for breast-feeding at 6 months is formula only; age and income are continuous.

In the adjusted models, we additionally observed racial/ethnic differences for factors 1 (concern about infant undereating or becoming underweight) and 4 (concern about infant overeating or becoming overweight). Mothers classified as Hispanic/Latina or other reported greater concern about their infant undereating or becoming underweight than non-Hispanic White mothers (*β*: 0·25 (95 % CI 0·04, 0·46) and *β*: 0·24 (95 % CI 0·01, 0·46), respectively), while Hispanic/Latina and Black mothers reported greater concern about their infant overeating or becoming overweight than non-Hispanic White mothers (*β*: 0·25 (95 % CI 0·01, 0·49) and *β*: 0·31 (95 % CI 0·01, 0·61), respectively).

## Discussion

In this secondary data analysis with a high-risk sample, racial/ethnic minority mothers reported feeding practices and beliefs that demonstrated greater concern about their infant’s hunger, as well as the amount of food their infant consumed (i.e. concern about under- and overeating) and his or her weight status (i.e. concern about the infant becoming underweight or overweight) compared with non-Hispanic White mothers at 6 months postpartum. Racial/ethnic minority mothers were also more likely to feed their infant on a schedule and less likely to socially interact with their infant while feeding, than White mothers. Observed differences were independent of maternal age, marital status, parity, education, household income and breast-feeding status.

The findings from this study are consistent with the previous literature on racial/ethnic differences in maternal feeding practices in early childhood^(13,14)^. Perrin *et al.* found that racial/ethnic minority mothers, especially Hispanics/Latinas, were more likely to encourage their infant to finish feedings^(13)^. Similarly, in this study, racial/ethnic minority mothers expressed greater concern regarding underweight or undereating. However, unlike Perrin *et al*., feeding as a first response to a crying or fussy baby did not differ by race/ethnicity in this sample. Our finding that racial/ethnic minority mothers were more likely to feed their infant on a schedule is consistent with findings by Taveras *et al.*, in which Hispanic/Latina and Black mothers had nearly two times the odds of engaging in restrictive feeding practices compared with White mothers^(14)^. This study advances the literature by examining and observing differences in additional maternal feedings practices and beliefs (e.g. concern about infant overeating or becoming overweight and social interaction during feeding), across race/ethnicity. Our findings suggest that mothers, especially racial/ethnic minorities, may simultaneously be concerned about their infant under- and overeating or becoming under- or overweight, and that social interaction during feeding, which may foster responsive feeding, varies across groups.

Differences with regard to a mother’s concern about her infant being hungry or becoming underweight may explain why racial/ethnic minority infants are more likely to be introduced to solids early^(13,14)^. Further underscoring this point, we found that racial/ethnic minority mothers were more likely to mix cereal in their infant’s bottle so he or she would stay full longer and felt as though their infant wanted more than just formula and/or breast milk prior to 4 months of age. This is an important topic for future intervention efforts to consider as early introduction to solids (i.e. prior to 4 months of age) and may increase obesity risk^(25,26)^. The presence of significant differences across race/ethnicity in regard to feeding on a schedule may be another important intervention target as responsive feeding has long been recommended to foster the development of infant self-regulation, though more research explicitly measuring dimensions of appetite regulation (e.g. hunger, satiety and satiation) is needed^(10,27)^. Our findings also suggest that racial/ethnic minority mothers are relatively concerned about their infant’s weight status and/or weight gain trajectory. Thus, interventions targeting maternal feeding should address concerns about both infant’s undereating or becoming underweight and overeating or becoming overweight.

In targeting maternal infant feeding practices and beliefs, interventions must consider the contextual factors that shape those practices and beliefs. In addition to being influenced by factors related to socio-economic status and family structure, maternal infant feeding practices are also shaped by cultural influences^(28)^. For example, some racial/ethnic minorities prefer chubby infants and toddlers and frequently do not identify their overweight children as being overweight^(29)^. Cultural preferences in which heavier infants are perceived as healthier infants may shape the way mothers feed their children. Cultural preferences like these can also be exacerbated by life experiences like food insecurity; for example, one study described how Latina immigrant mothers not only needed to resist indulgent feeding practices but they also need to resist the pressure to give in that arises from the scarcity they faced during their own childhood^(30)^. Cultural preference and experiences like these likely contribute to the higher rates of racial/ethnic minority mothers engaging in feeding practices that increase obesity risk such as encouraging their infant to finish their bottle, supplemental feeding, bottle propping, immediately feeding their infant when crying and putting their infant to bed with a bottle^(12,13)^. Without consideration for cultural preferences and beliefs, intervention efforts aimed at promoting certain maternal feeding behaviours (e.g. nutrition counselling) are unlikely to be successful.

The findings from this study also highlight additional practices and beliefs interventions, particularly interventions targeted at high-risk low-income samples, should consider. Mothers in this sample frequently reported worrying about whether the amount of food their infant was eating was sufficient or healthy. Prior research has found that low-income mothers are more concerned about their infant’s hunger than high-income mothers and that they may find it difficult to withhold food from their children even when they have just eaten^(18,31)^. The majority of mothers in this sample also reported that feeding their infant was the first thing they would do when he or she was fussy. Low-income mothers may thus require additional support around picking up on infant hunger and satiety cues and calming without feeding. Low-income mothers are less likely to believe that infants know their own hunger and satiety than high-income mothers^(32)^; thus, interventions require teaching or other encouragement of mothers about the ability of infants to self-regulate.

Taken together, the findings from this study may be used to inform maternal infant feeding intervention efforts aimed at high-risk populations. Additional efforts in this area are paramount as maternal feeding practices during infancy have been associated with later feeding practices, eating behaviours and obesity risk^(33–35)^. For example, infants who are encouraged to finish their bottles have been shown to be about twice as likely to eat all of the food on their plate at 6 years old than those who were rarely encouraged to finish their bottle during early infancy^(34)^. Further, young children from both racial/ethnic minority backgrounds and low socio-economic status households have a disproportionate risk for obesity and are more likely to be exposed to feeding practices associated with weight gain^(36)^.

While the present study expands the literature on racial/ethnic differences in the ways in which mothers feed their infants, it is not without limitations. First, as a cross-sectional secondary data analysis, we were limited in the analyses we could conduct and variables we could adjust for. Also, the fact that mothers self-reported their feeding practices means our results may be biased by mothers’ perception. Additional research using more robust study designs and measures would further expand the literature on this topic and allow investigations of changes over time. Second, we classified race/ethnicity using the Department of Finance method^(23)^. Racial/ethnic classification is a complex task due to the heterogeneity and social and cultural complexity of groups, and method of classification can have important implications for the results. While prior studies used the rarest group method to classify multiracial individuals^(23)^, the majority of multiracial participants in this sample did not further specify their racial/ethnic background; thus, we included individuals identifying as multiracial in the ‘other’ category. Results from this group must be interpreted with caution due to extreme within-group heterogeneity. Finally, the study sample was comprised of low-income and smoke-exposed women living in the Providence area, RI; thus, our results may not be generalisable to broader populations. While the implications of smoke exposure on the relationship between race/ethnicity and maternal infant feeding are unknown, racial/ethnic differences in maternal infant feeding practices are likely more pronounced in samples that represent different levels of socio-economic status given associations between race/ethnicity, socio-economic status and infant feeding behaviours.

## Conclusions

This study revealed differences in maternal infant feeding practices and beliefs across race/ethnicity among low-income mothers at 6 months postpartum. These differences were not explained by mothers’ age, marital status, parity or educational level, nor by household income or breast-feeding status. Given the evidence linking maternal feeding practices during infancy to childhood obesity risk, interventions are needed to further promote responsive feeding among mothers identifying as racial/ethnic minorities and address their concerns regarding their infant’s hunger, amount of food being eaten and weight status.
